# Supplementary material for: Spatial Distribution and Temporal Dynamics of Candidatus Liberibacter Asiaticus in Different Stages of Embryos, Nymphs and Adults of Diaphorina citri
Source: Int J Mol Sci. 2023 May 19;24(10):8997. doi: 10.3390/ijms24108997 (PMC10219130; doi:10.3390/ijms24108997)
Supplement: Supplementary file 1 [file ijms-24-08997-s001.zip › ijms-2343073-supplementary.pdf]

Supplemental Information

Spatial distribution and temporal dynamics of *Candidatus* Liberibacter asiaticus in different stages of embryos, nymphs and adults of *Diaphorina citri*

Xiaoge Nian, Shujie Wu, Jiayun Li, Yaru Luo, Jielan He, Shijian Tan, Desen Wang, Yijing Cen, Yurong He

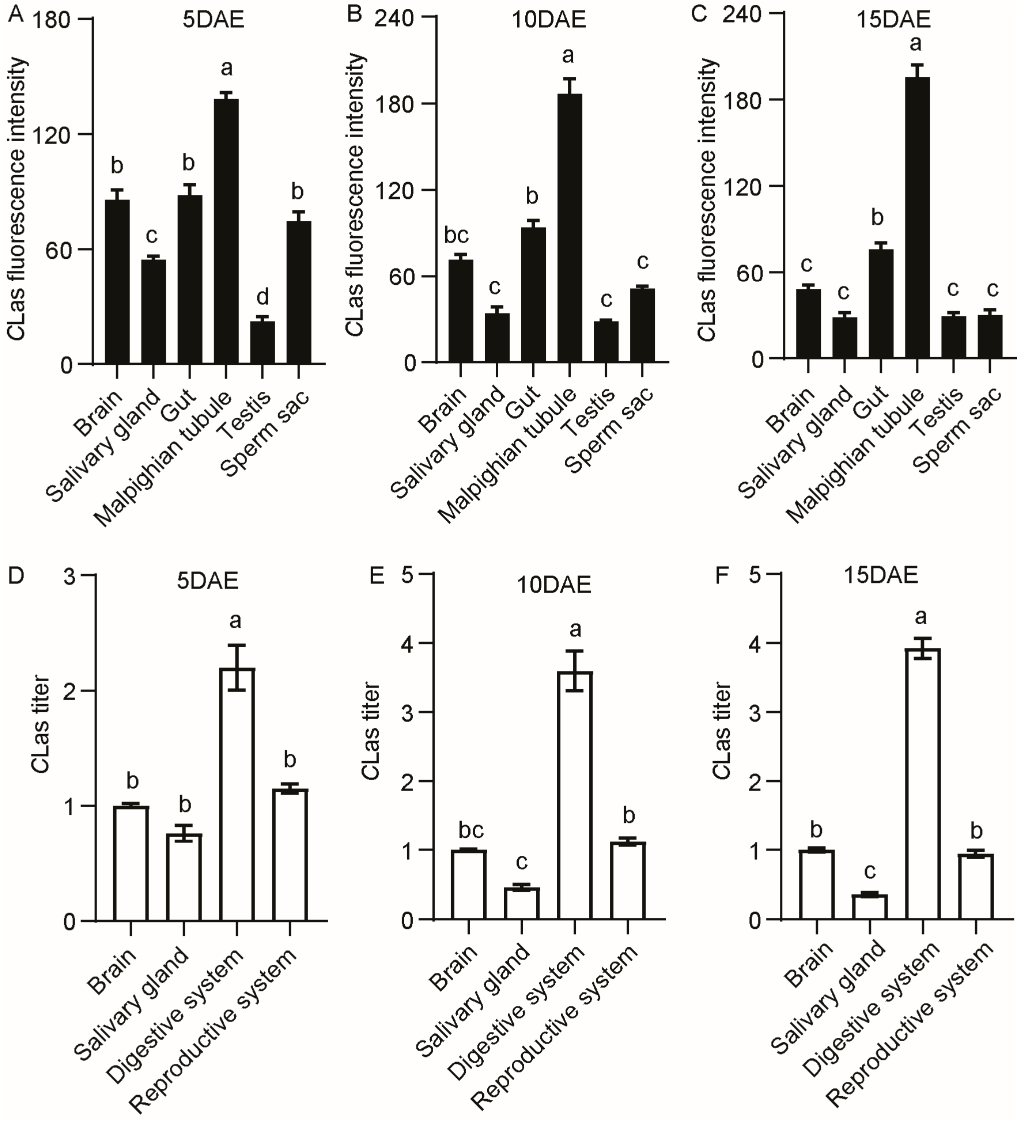

Figure S1. Comparison of fluorescence intensity and relative *C*Las titer in the different tissues of *C*Las-positive adult male at 5 DAE, 10 DAE, and 15 DAE. (**A-C**) *C*Las fluorescence intensity in the different tissues of male at 5 DAE, 10 DAE, and 15 DAE, respectively. **(D-E)** Relative *C*Las titer in the different tissues of male at 5 DAE, 10 DAE, and 15 DAE, respectively.

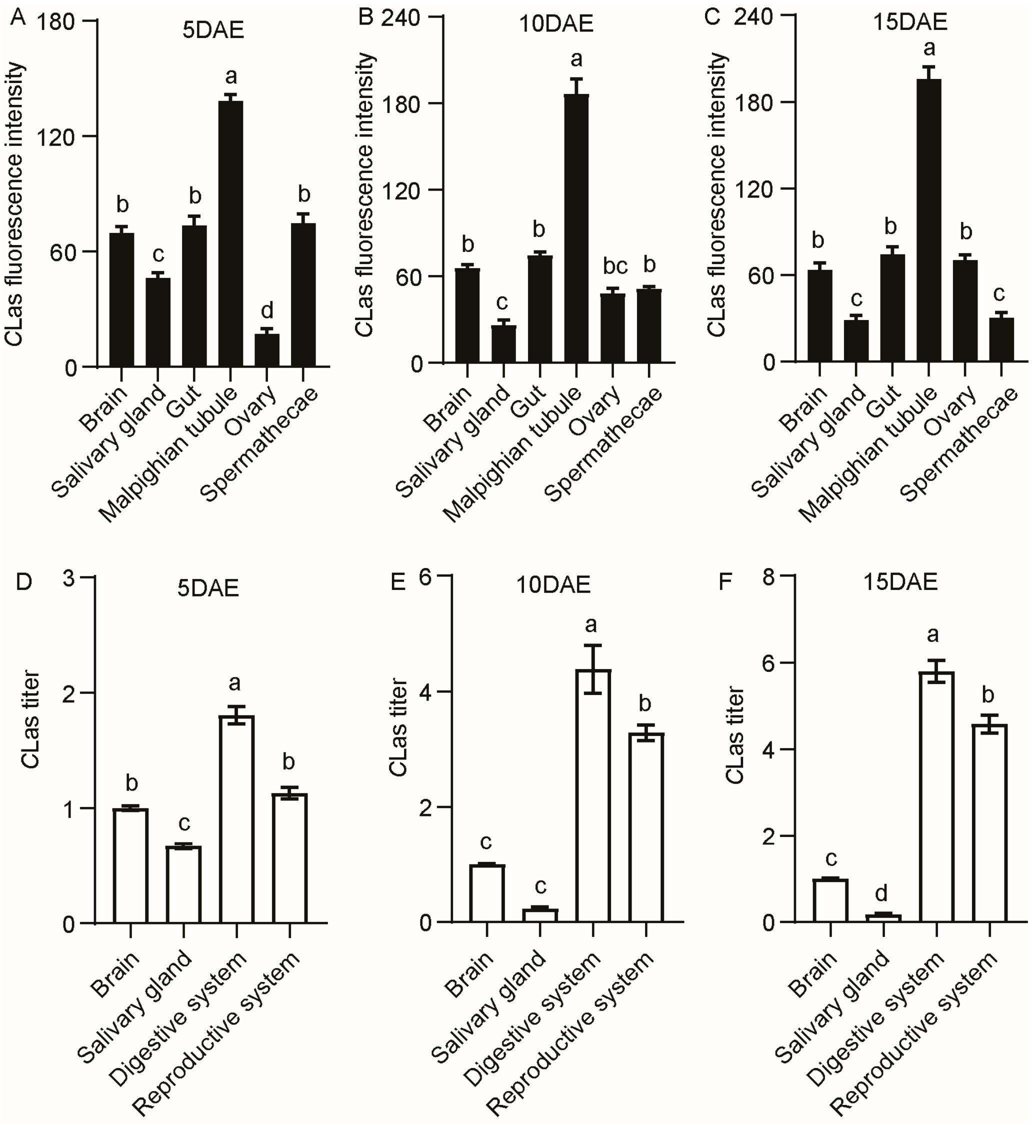

Figure S2. Comparison of fluorescence intensity and relative *C*Las titer in the different tissues of *C*Las-positive adult female at 5 DAE, 10 DAE, and 15 DAE. (**A-C**) *C*Las fluorescence intensity in the different tissues of female at 5 DAE, 10 DAE, and 15 DAE, respectively. **(D-E)** Relative *C*Las titer in the different tissues of female at 5 DAE, 10 DAE, and 15 DAE, respectively.
